# ANXA2 is a potential biomarker for cancer prognosis and immune infiltration: A systematic pan-cancer analysis

**DOI:** 10.3389/fgene.2023.1108167

**Published:** 2023-01-12

**Authors:** Yijie Ning, Yufei Li, Hongqin Wang

**Affiliations:** ^1^ Department of Neurosurgery, The First Hospital of Shanxi Medical University, Taiyuan, China; ^2^ Department of Neurosurgery, The Second Hospital of Shanxi Medical University, Taiyuan, China

**Keywords:** ANXA2, biomarker, immune infiltration, pan-cancer, prognosis

## Abstract

**Background:** Annexin A2 (ANXA2) belongs to the Annexin A family and plays a role in epithelial-mesenchymal transition, fibrinolysis, and other physiological processes. Annexin A2 has been extensively implicated in tumorigenesis and development in previous studies, but its precise role in pan-cancer remains largely unknown.

**Methods:** We adopted bioinformatics methods to explore the oncogenic role of Annexin A2 using different databases, including the Cancer Genome Atlas (TCGA), the Genotype-Tissue Expression (GTEx) biobank, the Human Protein Atlas (HPA), the Gene Expression Profiling Interaction Analysis (GEPIA) and cBioPortal. We analyzed the differential expression of Annexin A2 in different tumors and its relationship with cancer prognosis, immune cell infiltration, DNA methylation, tumor mutation burden (TMB), microsatellite instability (MSI) and mismatch repair (MMR). Furtherly, we conducted a Gene Set Enrichment Analysis (GSEA) to identify the Annexin A2-related pathways.

**Results:** Annexin A2 expression was upregulated in most cancers, except in kidney chromophobe (KICH) and prostate adenocarcinoma (PRAD). Annexin A2 showed a good diagnostic efficacy in twelve types of cancer. The high expression of Annexin A2 was significantly associated with a reduced overall survival, disease-specific survival and progression-free interval in seven cancers. The Annexin A2 expression was variably associated with infiltration of 24 types of immune cells in 32 tumor microenvironments. In addition, Annexin A2 expression was differently associated with 47 immune checkpoints, immunoregulators, DNA methylation, tumor mutation burden, microsatellite instability and mismatch repair in pan-cancer. Gene Set Enrichment Analysis revealed that Annexin A2 was significantly correlated with immune-related pathways in fifteen cancers.

**Conclusion:** Annexin A2 widely correlates with immune infiltration and may function as a promising prognostic biomarker in many tumors, showing its potential as a target for immunotherapy in pan-cancer.

## Introduction

After cardiovascular diseases, cancer is the second leading cause of death worldwide (Myerson et al., 2019; Sung et al., 2021; Siegel et al., 2022). About 28.4 million new cancer cases are expected to be diagnosed in 2040, placing a heavy burden on the healthcare systems of various countries (Sung et al., 2021). Despite a better understanding of cancer and novel treatments such as immunotherapy (Singh and McGuirk, 2020), cancer remains difficult to cure (Sung et al., 2021; Siegel et al., 2022). There is a need to find suitable biomarkers to guide treatments and predict clinical outcomes. The pan-cancer analysis of genes detects molecular abnormalities at the DNA, RNA, protein and epigenetic levels. As a result, it is possible to identify commonalities, differences, and emerging themes in tumor lineages (Weinstein et al., 2013).

Annexin A2 (ANXA2) is a calcium-dependent phospholipid binding protein and regulates cellular growth (Hajjar and Krishnan, 1999; Wang and Lin, 2014). ANXA2 is involved in various pathophysiological processes, including epithelial-mesenchymal transition, fibrinolysis and cancer drug resistance (Wang et al., 2019; Huang et al., 2022). *In vitro*, ANXA2 promoted migration and invasion of esophageal squamous carcinoma cells by activating the MYC-HIF1A-VEGF signaling cascade (Wu et al., 2019). Similar findings have been observed in liver cancer stem cells through the miR-101/ANXA2/EGR1 regulatory pathway (Ma et al., 2021a). Also, ANXA2 promoted gastric cancer cell invasion and metastasis through the EphA2-YES1-ANXA2 signaling pathway (Mao et al., 2022). ANXA2 may play a role in a number of non-neoplastic conditions, such as autoimmune diseases, thrombosis, hemorrhagic disorders and viral infections (Huang et al., 2022). ANXA2 is considered an autoantigen of autoimmune disorders like lupus nephritis, antiphospholipid syndrome and Behcet’s disease (Caster et al., 2015; Hussain et al., 2018; Müller-Calleja and Lackner, 2018). In addition, ANXA2 is closely related to immune regulation. Specific binding of ANXA2 and toll-like receptor 2 (TLR2) induces dendritic cell differentiation and maturation, increases the expression of CD80 and CD86 and favors antigen presentation by the major histocompatibility complex (MHC) class I pathway (Andersen et al., 2016).

Owing to the deficiency of relevant pan-cancer research and the important function of ANXA2 in tumors, a comprehensive pan-cancer analysis of ANXA2 were conducted using various databases. We revealed the relationship between the expression of ANXA2 and the prognosis, immune infiltration and genetic alterations of 33 types of cancer. In addition, we explored the involved signaling pathways. Our results indicated that ANXA2 may be a promising immune-related prognostic biomarker in a variety of cancers. This study may provide new insights into the application of ANXA2 in tumor immunotherapy.

## Materials and methods

### Data processing

Transcriptome and clinical data from 33 cancers were downloaded from TCGA database (https://tcgadata.nci.nih.gov/tcga/) using UCSC Xena (https://xena.ucsc.edu/), a genome browser for visualizing gene and variant information. The 33 tumor datasets from the TCGA database were obtained by clicking on the “Launch Xena” and “DATA SETS” options on the UCEC website. Gene expression and clinical data from any tumor can be downloaded in the “gene expression RNAseq” and “phenotype” sections of the tumor datasets, respectively. We further downloaded the RNA sequencing data of 31 different tissues from the GTEx biobank (https://commonfund.nih.gov/GTEx) to obtain more normal tissues. We clicked on “Downloads”—“Open Access Data” from the GTEx database homepage. GTEx Analysis V8 (dbGaP Accession phs000424.v8.p2) version was chosen. The annotation file and gene expression data were downloaded in the “Annotations” and “RNA-Seq Data” sections, respectively. The data from TCGA and GTEx was merged. The RNA sequencing data was transformed into transcripts per million (TPM) reads and normalized by log2 transformation for the following analysis.

### Differential expression analysis and immunohistochemistry staining

An analysis of ANXA2 expression in tumor and normal tissues was performed using R statistical software (version 4.0.3). Box plots were created using the R package “ggplot2”. The HPA database (https://www.proteinatlas.org/) was used to investigate the gene expression of ANXA2 in 27 types of normal tissues, the protein expression of ANXA2 in 20 cancers and the protein location of ANXA2 in cells. We downloaded immunohistochemical images of colon adenocarcinoma (COAD), glioblastoma multiforme (GBM), liver hepatocellular carcinoma (LIHC), prostate adenocarcinoma and stomach adenocarcinoma (STAD) with the corresponding normal tissues from HPA to evaluate the differential expression of ANXA2. The GEPIA database (http://gepia.cancer-pku.cn/) was used to verify the differential expression of ANXA2 in the aforementioned tumors and corresponding normal tissues.

### Diagnostic and prognostic analysis

The receiver operating characteristic (ROC) curve was calculated to evaluate the diagnostic ability of ANXA2 by the R software (version 4.0.3). The curves were drawn using the R packages “pROC” and “ggplot2”. The area under the curve (AUC) > .8 was considered of adequate diagnostic value (Mandrekar, 2015). The survival analysis was conducted to analyze the prognosis of differential expression of ANXA2 in 33 types of cancer by using the packages “survival” and “survminer”. The overall survival (OS), disease-specific survival (DSS) and progression-free interval (PFI) were used to evaluate the prognostic value of ANXA2. The Kaplan-Meier method (Kaplan-Meier curve plus univariate Cox regression analysis) was used for survival analysis. Also, a pan-cancer survival analysis of ANXA2 gene was performed using the GEPIA database to explore the prognostic value of ANXA2 in all 33 tumors from the TCGA database.

### Relationship between ANXA^2^ and tumor staging

The correlation between ANXA2 expression and tumor staging was tested with the R software (version 4.0.3). Box diagrams were drawn using the R package “ggplot2”.

### Nomogram construction and evaluation

Tumor types with statistical significance in both survival analysis and tumor staging correlation analysis were chosen to draw the nomograms using R statistical software (version 4.0.3), so as to highlight the value of ANXA2 expression in cancer prognosis and reduce the interference of other clinical factors. R package “rms” and “survival” were used to construct nomograms and calibration curves which were used to verify the accuracy of the nomograms.

### Relationship between ANXA2 and immunity

The RNA sequencing expression profile was used to investigate the infiltration of 24 types of immune cells in tumors with the R software (version 4.0.3). The correlation between ANXA2 expression and immune cell infiltration in 33 types of cancer was analyzed by the R package “GSVA”. The correlation between ANXA2 expression and StromalScore, ImmuneScore and ESTIMATEScore was analyzed by the R package “estimate”, |r| > .3 was considered statistically relevant. In addition, we conducted a co-expression analysis to explore the correlation between expression of ANXA2 and 47 immune checkpoints and immunomodulators (including immune-activating genes, immune-suppressing genes, chemokine ligands, chemokine receptors and MHC genes) by using the R package “ggplot2”. The analyses were performed by using the R software (version 4.0.3).

### Relationship among ANXA2, DNA methylation and genetic alterations

The correlation analysis was performed to investigate the relationship between ANXA2 expression and DNA methylation, TMB, MSI and MMR by the R software (version 4.0.3). The *p*ackage “ggplot2” was used for visualizing data. The DNA methylation analysis was based on Illumina methylation 450 data and cg02395965 probe. The cBioPortal website (http://www.cbioportal.org/) was applied to analyze the genetic characteristics of ANXA2 in 30 types of cancer. The genetic variation characteristics of ANXA2 included mutation, structural variants, amplification, deep deletion and multiple alterations in pan-cancer.

### Gene set enrichment analysis

The Gene Set Enrichment Analysis (GSEA) was performed to predict the ANXA2-related signaling pathways by using the R software (version 4.0.3). The package “clusterProfiler” was used to obtain the enrichment maps. The adjusted *p*-value (<.05), normalized enrichment score (|NES| > 1), and False Discovery Rate (FDR, q value < .25) were used as criteria to determine a statistically significant phenotype.

### Statistical analysis

The differential expression of ANXA2 was estimated using the Wilcoxon rank sum testand Wilcoxon signed rank test. A log-rank test was used to perform the survival analysis of ANXA2. The Kruskal–Wallis test was used to conduct the correlation analysis between ANXA2 expression and tumor TNM staging. The Spearman’s rank correlation coefficient was used to estimate the correlation between ANXA2 expression and infiltration of 24 types of immune cells, expression of 47 immune checkpoints, StromalScore, ImmuneScore, ESTIMATEScore, immunomodulators, DNA methylation, TMB, MSI and MMR. A *p*-value < .05 was considered statistically significant (*p < .05, **p < .01, ***p < .001).

## Results

### Expression levels of ANXA2

**FIGURE 1:**
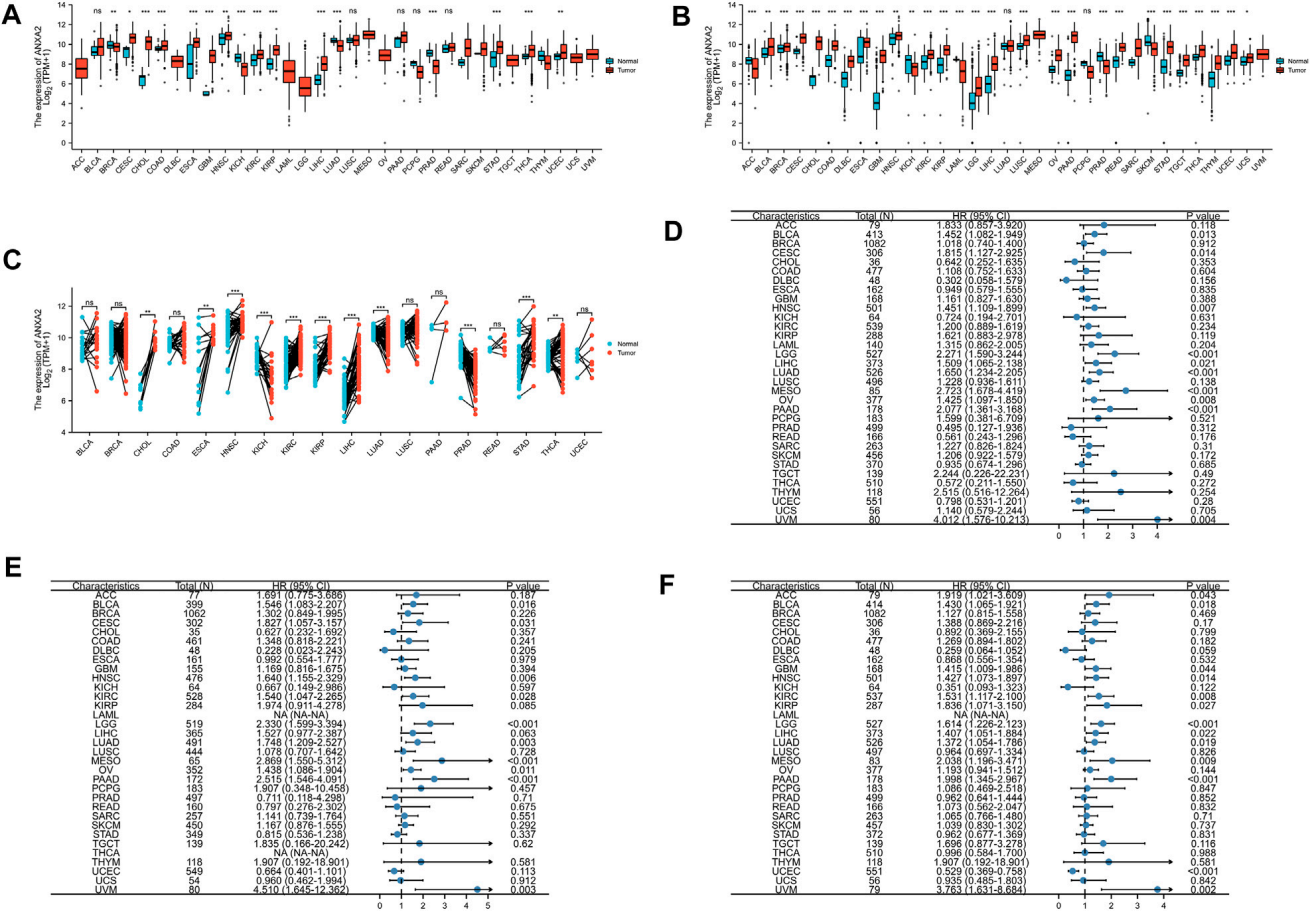
Differential expression and prognostic value of ANXA2 in pan-cancer. **(A)** Expression of ANXA2 gene in 33 cancers based on the data from the TCGA database. **(B)** Expression of ANXA2 gene in 33 cancers based on the data from TCGA and GTEx databases. **(C)** Expression of ANXA2 gene in paired samples based on the data from the TCGA database. Forest plot of **(D)** OS, **(E)** DSS and **(F)** PFI associated with ANXA2 expression in 33 types of cancer. *p < .05, **p < .01 and ***p < .001.

In this study, we aimed at analyzing the expression of ANXA2 in various tumors. The sample sizes for each comparison were added to Supplementary Table S1. Based on the TCGA database, the expression of ANXA2 in cervical squamous cell carcinoma (CESC), CHOL, COAD, esophageal carcinoma (ESCA), GBM, head and neck squamous cell carcinoma (HNSC), kidney renal clear cell carcinoma (KIRC), kidney renal papillary cell carcinoma (KIRP), LIHC, STAD, thyroid carcinoma (THCA) and uterine corpus endometrial carcinoma (UCEC) was higher compared with corresponding normal tissues. The expression of ANXA2 in breast invasive carcinoma (BRCA), KICH, lung adenocarcinoma (LUAD) and PRAD was lower compared with corresponding normal tissues (Figure 1A). Due to the shortage of normal samples in the TCGA database, we combined the data of TCGA and GTEx databases for expression analysis. Compared with normal tissues, ANXA2 was upregulated in bladder urothelial carcinoma (BLCA), BRCA, CESC, CHOL, COAD, lymphoid neoplasm diffuse large B cell lymphoma (DLBC), ESCA, GBM, HNSC, KIRC, KIRP, brain lower grade glioma (LGG), LIHC, lung squamous cell carcinoma (LUSC), ovarian serous cystadenocarcinoma (OV), pancreatic adenocarcinoma (PAAD), rectum adenocarcinoma (READ), STAD, testicular germ cell tumor (TGCT), THCA, thymoma (THYM), UCEC and uterine carcinosarcoma (UCS). ANXA2 was downregulated in adrenocortical carcinoma (ACC), KICH, acute myeloid leukemia (LAML), PRAD and skin cutaneous melanoma (SKCM) (Figure 1B). In paired samples from TCGA database, ANXA2 was upregulated in CHOL, ESCA, HNSC, KIRC, KIRP, LIHC, STAD, THCA compared with normal tissues. ANXA2 was downregulated in KICH, LUAD, PRAD compared with normal tissues (Figure 1C). Based on the TCGA database alone, the expression of ANXA2 was lower in BRCA compared with corresponding normal tissues, the average expression levels of ANXA2 were 9.675 and 9.887 in tumor and normal tissues, respectively. When TCGA and GTEx databases were combined together, the expression of ANXA2 was higher in BRCA compared with corresponding normal tissues, the average expression levels of ANXA2 were 9.675 and 9.545 in tumor and normal tissues, respectively.

**FIGURE 2:**
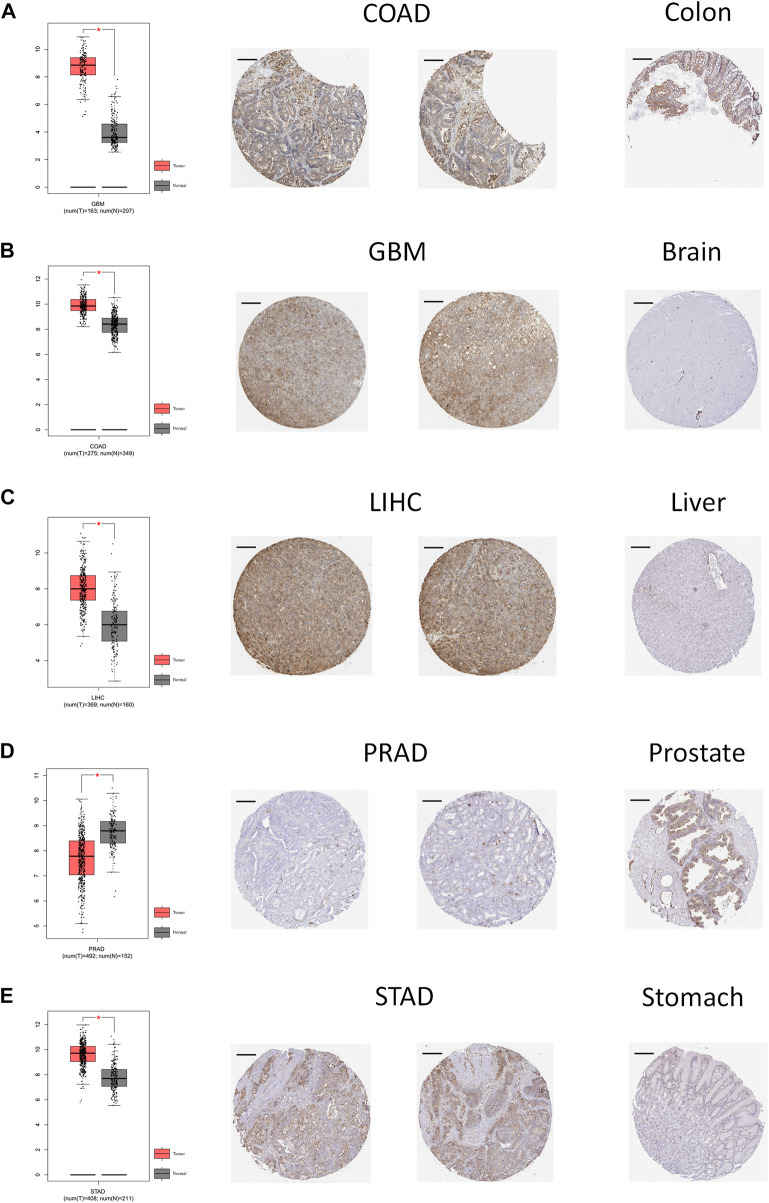
Comparison of ANXA2 gene expression between tumor and normal tissues (left) and immunohistochemistry images in tumor (middle) and normal (right) tissues. Comparison of ANXA2 gene expression between **(A)** COAD and colon, **(B)** GBM and brain, **(C)** LIHC and liver, **(D)** PRAD and prostate and **(E)** STAD and stomach. Scale bar = 200 μm. *p < .05.

We further investigated the gene and protein expression levels of ANXA2 through the HPA database. Among 27 normal tissues, protein expression level of ANXA2 was highest in the esophagus (Supplementary Figure S1A). Among 20 different types of cancer, the protein level of ANXA2 was highest in renal cell carcinoma (Supplementary Figure S1B). We speculated that ANXA2 is more likely to exert significant physiological or pathological functions in normal tissues and cancers with high levels of ANXA2 protein expression. The ANXA2 protein was mainly located in the plasma membrane and cytoplasmic matrix (Supplementary Figure S1C). To evaluate the expression of ANXA2 in more detail, we analyzed the immunohistochemistry results obtained through the HPA database. In addition, we compared the results with the ANXA2 gene expression from the GEPIA database. The findings were consistent between the two databases (Figures 2A–E). Normal brain, liver and stomach tissues showed a weak or no ANXA2 staining, whereas GBM, LIHC and STAD tissues showed a moderate staining. Normal colon tissues showed a moderate staining, while COAD tissues showed a strong staining. Normal prostate tissues showed a strong staining, while PRAD tissues showed a weak or no staining.

### The diagnostic value of ANXA2

**FIGURE 3:**
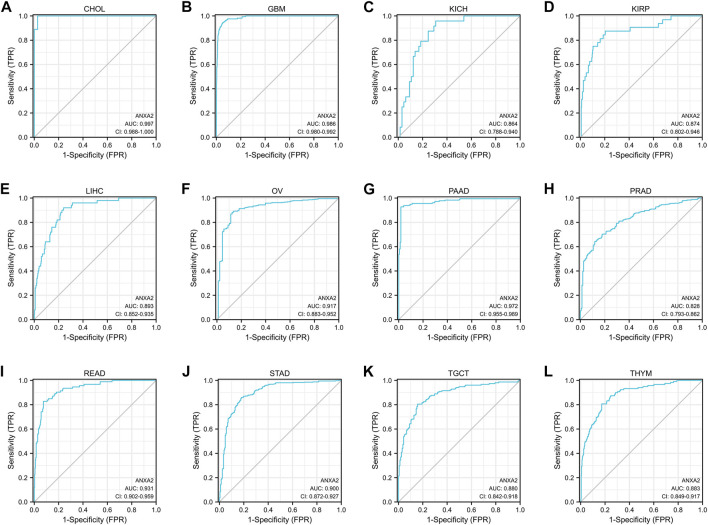
The area under the curve of ROC curves verified the diagnostic ability of ANXA2 in **(A–L)** CHOL, GBM, KICH, KIRP, LIHC, OV, PAAD, PRAD, READ, STAD, TGCT and THYM.

The ROC curve was used to evaluate the diagnostic efficacy of ANXA2 in 33 types of cancer. ANXA2 showed a high diagnostic efficiency (AUC > .8) in the following 12 types of cancer: CHOL (AUC = .997), GBM (AUC = .986), KICH (AUC = .864), KIRP (AUC = .874), LIHC (AUC = .893), OV (AUC = .917), PAAD (AUC = .972), PRAD (AUC = .828), READ (AUC = .931), STAD (AUC = .900), TGCT (AUC = .880) and THYM (AUC = .883) (Figures 3A–L).

### The prognostic value of ANXA2

**FIGURE 4:**
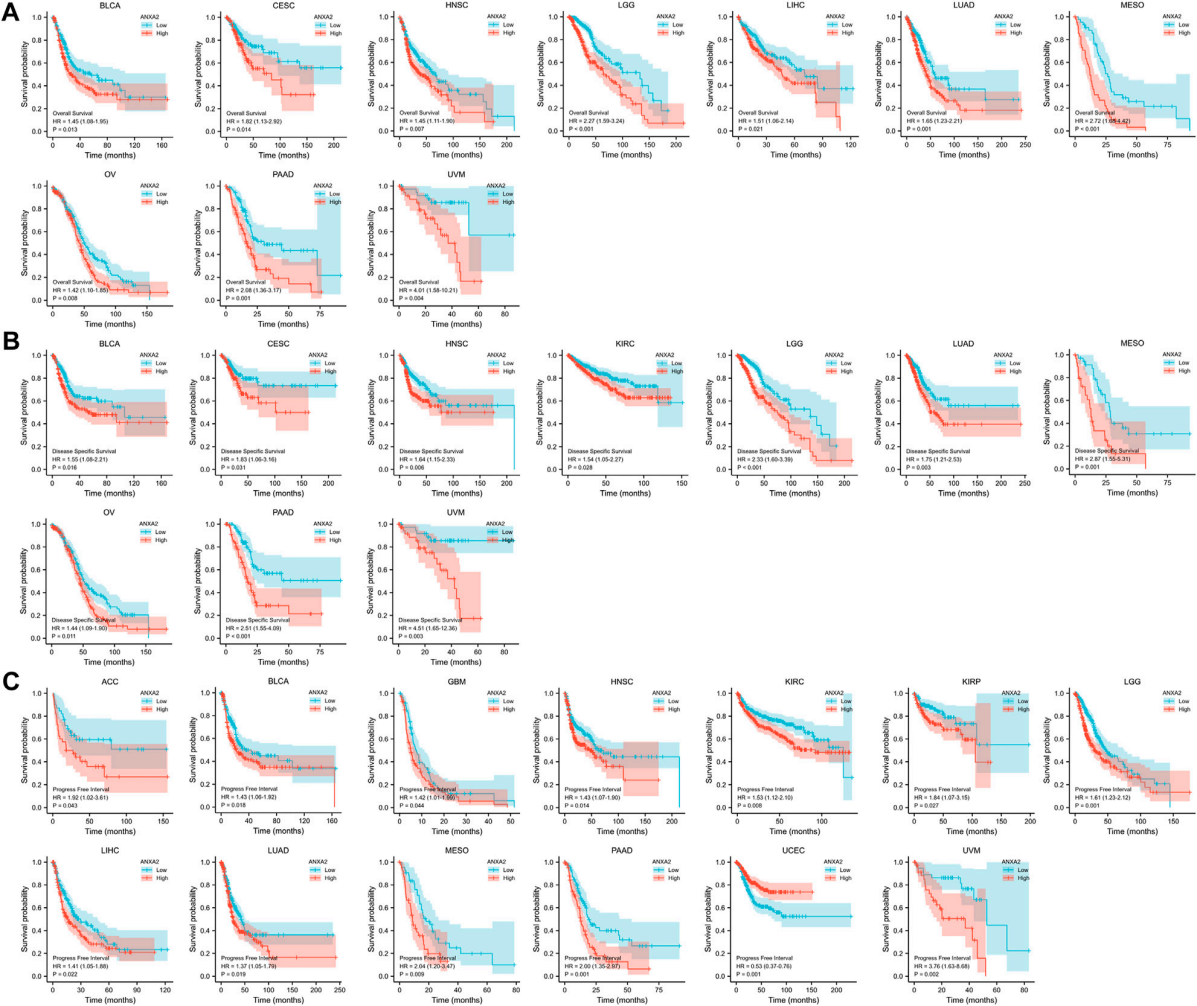
Kaplan-Meier analysis of the association between ANXA2 expression and **(A)** OS, **(B)** DSS and **(C)** PFI.

A survival analysis was conducted to examine the association between ANXA2 expression and prognosis of 33 types of cancer. Based on the Cox proportional hazards model, the ANXA2 expression level were related to OS of BLCA (*p* = .013), CESC (*p* = .014), HNSC (*p* = .007), LGG (*p* < .001), LIHC (*p* = .021), LUAD (*p* < .001), mesothelioma (MESO) (*p* < .001), OV (*p* = .008), PAAD (*p* < .001) and uveal melanoma (UVM) (*p* = .004) (Figure 1D). The Kaplan-Meier survival analysis showed that the high expression of ANXA2 was associated with a short OS in the aforementioned tumors (Figure 4A). The expression level of ANXA2 correlated with DSS of BLCA (*p* = .016), CESC (*p* = .031), HNSC (*p* = .006), KIRC (*p* = .028), LGG (*p* < .001), LUAD (*p* = .003), MESO (*p* < .001), OV (*p* = .011), PAAD (*p* < .001) and UVM (*p* = .003) (Figure 1E). The Kaplan-Meier survival analysis showed that the high expression of ANXA2 was associated with a poor prognosis in the ten types of cancer described above (Figure 4B).

According to the forest plot, ANXA2 expression was related to PFI in ACC (*p* = .043), BLCA (*p* = .018), GBM (*p* = .044), HNSC (*p* = .014), KIRC (*p* = .008), KIRP (*p* = .027), LGG (*p* < .001), LIHC (*p* = .022), LUAD (*p* = .019), MESO (*p* = .009), PAAD (*p* < .001), UCEC (*p* < .001) and UVM (*p* = .002) (Figure 1F). The Kaplan-Meier survival analysis showed that the high expression of ANXA2 was associated with a poor PFI in ACC, BLCA, GBM, HNSC, KIRC, KIRP, LGG, LIHC, LUAD, MESO, PAAD and UVM. The low expression of ANXA2 was associated with a poor PFI in UCEC (Figure 4C). The pan-cancer survival analysis of ANXA2 gene based on the GEPIA database showed that high expression of ANXA2 was associated with lower overall survival in all 33 types of cancer (*p* = 0) (Supplementary Figure S2).

### Relationship between ANXA2 gene expression and tumor staging

**FIGURE 5:**
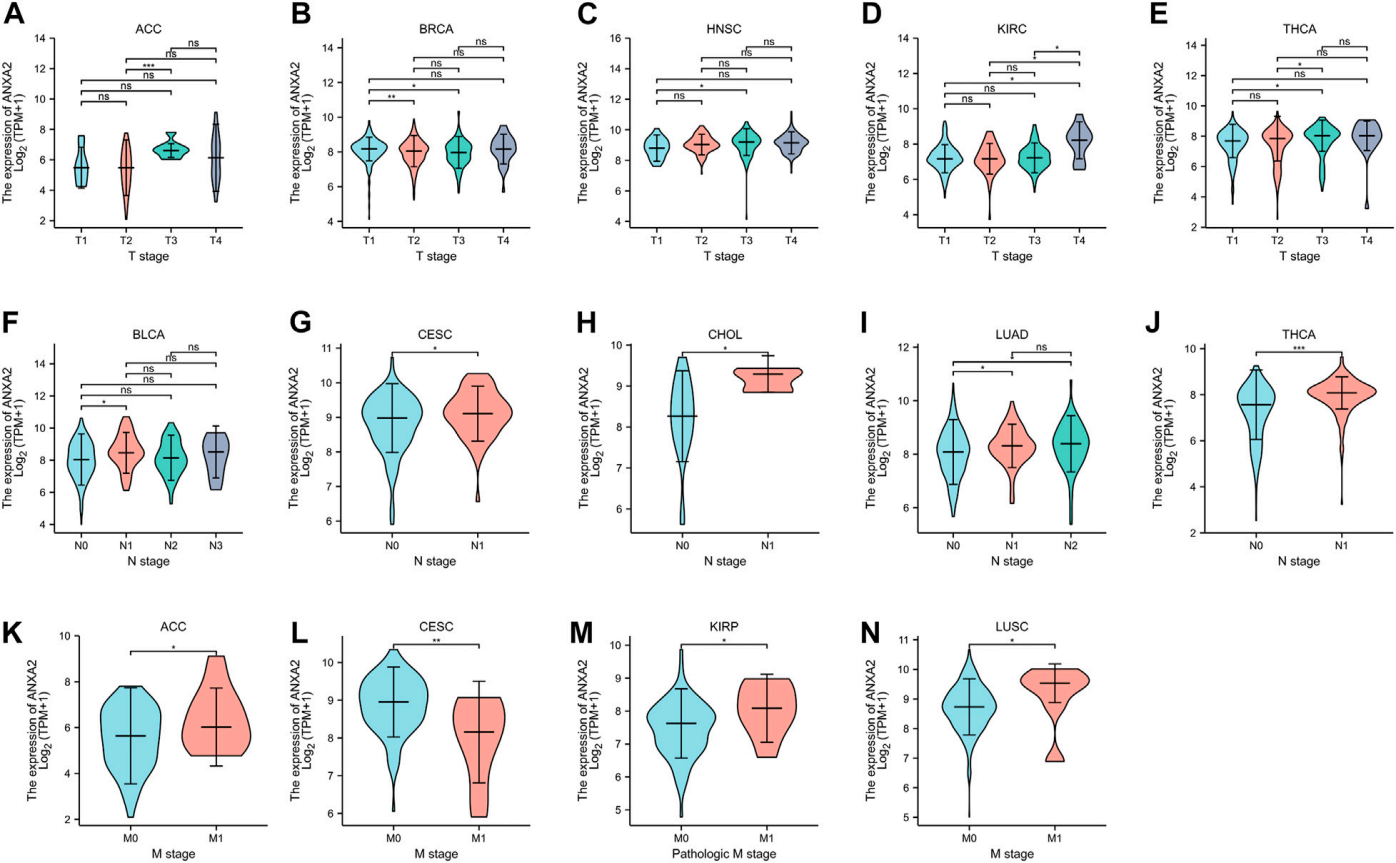
Relationship between ANXA2 expression and clinical TNM staging in pan-cancer. **(A–E)** Relationship between ANXA2 expression and clinical T stage in ACC, BRCA, HNSC, KIRC and THCA. **(F–J)** Relationship between ANXA2 expression and clinical N stage in BLCA, CESC, CHOL, LUAD and THCA. **(K–N)** Relationship between ANXA2 expression and clinical M stage in ACC, CESC, KIRP and LUSC. *p < .05, **p < .01 and ***p < .001.

The gene expression of ANXA2 was related to the clinical T stage of ACC, BRCA, HNSC, KIRC and THCA (Figures 5A–E), the clinical M stage of BLCA, CESC, CHOL, LUAD and THCA (Figures 5F–J) and the clinical N stage of ACC, CESC, KIRP and LUSC (Figures 5K–N) in 33 types of cancer.

### Nomogram construction and evaluation

BLCA, HNSC and LUAD were selected for the construction of nomograms. The clinical factors included in the nomogram for BLCA were: ANXA2 expression, age, clinical T, M, N stage and gender (Supplementary Figure S3A). The clinical factors included in the nomogram for HNSC were ANXA2 expression, age, clinical T, N stage, gender and radiation therapy (Supplementary Figure S3C). The clinical factors included in the nomogram for LUAD were ANXA2 expression, age, clinical T, N stage, gender and race (Supplementary Figure S3E). Among the three nomograms, ANXA2 showed the best prognostic prediction ability, and the calibration curves confirmed the accuracy of the nomograms (Supplementary Figures S3B, D, F). The nomograms further showed the good prognostic value of ANXA2 in different tumor types.

### Relationship between ANXA2 gene expression and tumor immune infiltration

**FIGURE 6:**
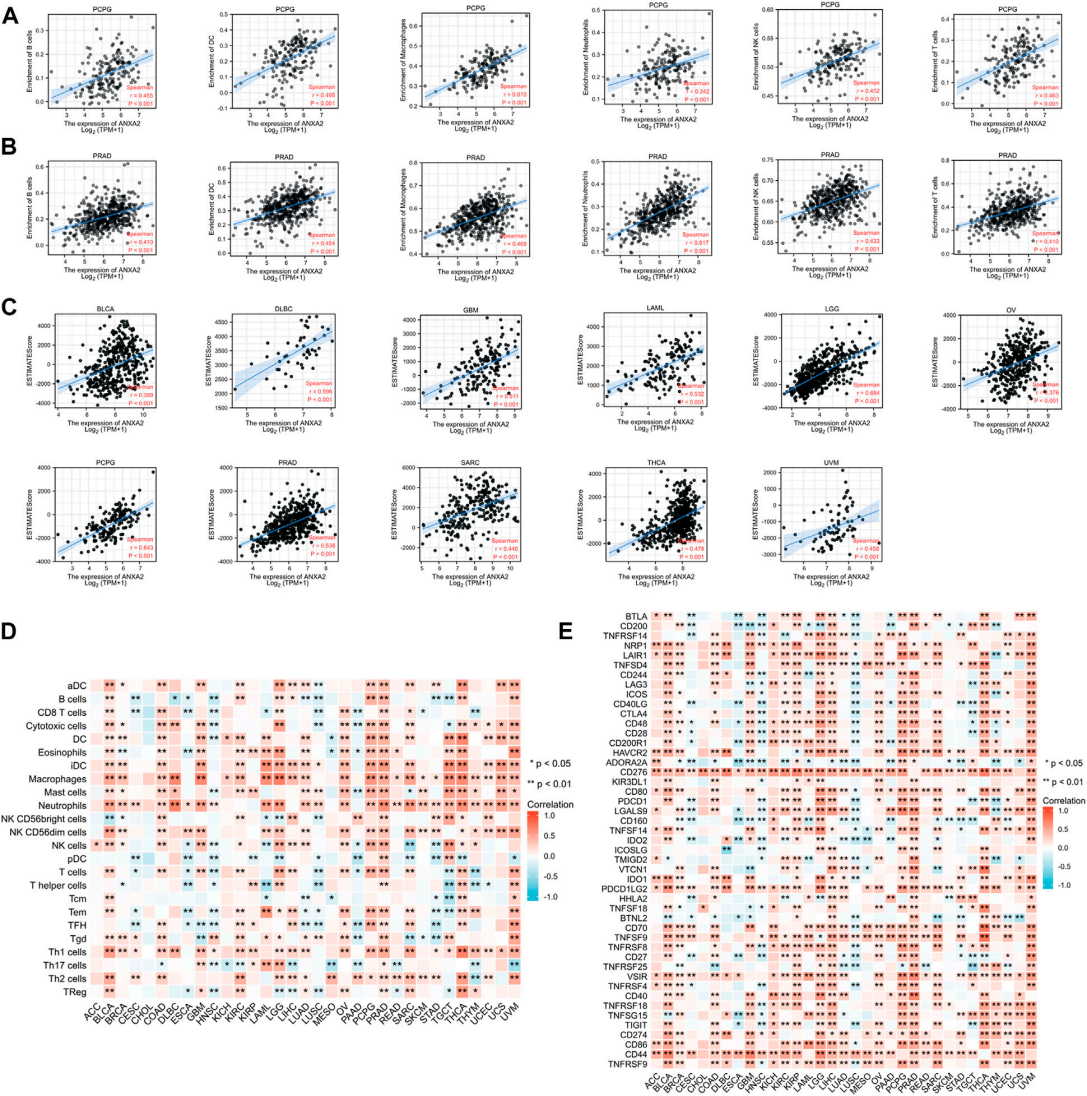
Correlation analysis of ANXA2 gene expression in immune cell infiltration, 47 immune checkpoints and ESTIMATEScore. **(A,B)** Correlation between ANXA2 gene expression and six immune cells (B cells, dendritic cells, macrophages, neutrophils, NK cells and T cells) in PCPG and PRAD. **(C)** ANXA2 expression significantly correlated with the ESTIMATEScore in 23 types of cancer. **(D)** Correlation between ANXA2 gene expression and infiltration of 24 types of immune cells in 33 types of cancer. **(E)** Correlation between ANXA2 gene expression and 47 immune checkpoints in 33 types of cancer. *p < .05 and **p < .01.

The Spearman’s correlation coefficient was used to indicate the association between the gene expression of ANXA2 and immune infiltration in 33 types of cancer. The association between ANXA2 expression and infiltration of six important immune cells (B cells, dendritic cells, macrophages, neutrophils, NK cells and T cells) was analyzed firstly. ANXA2 expression was positively correlated with the immune cells mentioned before in pheochromocytoma and paraganglioma (PCPG) (Figure 6A) and PRAD (Figure 6B) (all r > .3, all *p* < .05). We also found that the ANXA2 expression positively correlated with the StromalScore of 12 types of cancer (Supplementary Figure S4A) (all r > .3, all *p* < .05), the ImmuneScore of 11 types of cancer (Supplementary Figure S4B) (all r > .3, all *p* < .05) and the ESTIMATEScore of 11 types of cancer (Figure 6C) (all r > .3, all *p* < .05). We further explored the association between the ANXA2 gene expression and infiltration of 24 types of immune cells in pan-cancer. ANXA2 gene expression was associated with varying levels of immune infiltration in 32 types of cancer (except CHOL) (Figure 6D) according to the findings. We explored the correlation between ANXA2 expression and 47 immune checkpoints in pan-cancer. ANXA2 expression was variably related to the immune checkpoints in all 33 types of cancer, with the most relevant correlation being with CD276 (31/33) (Figure 6E). CHOL was associated with two immune checkpoints, CD276 and TNFSF18.

### Relationship between ANXA2 gene expression and immunomodulators

**FIGURE 7:**
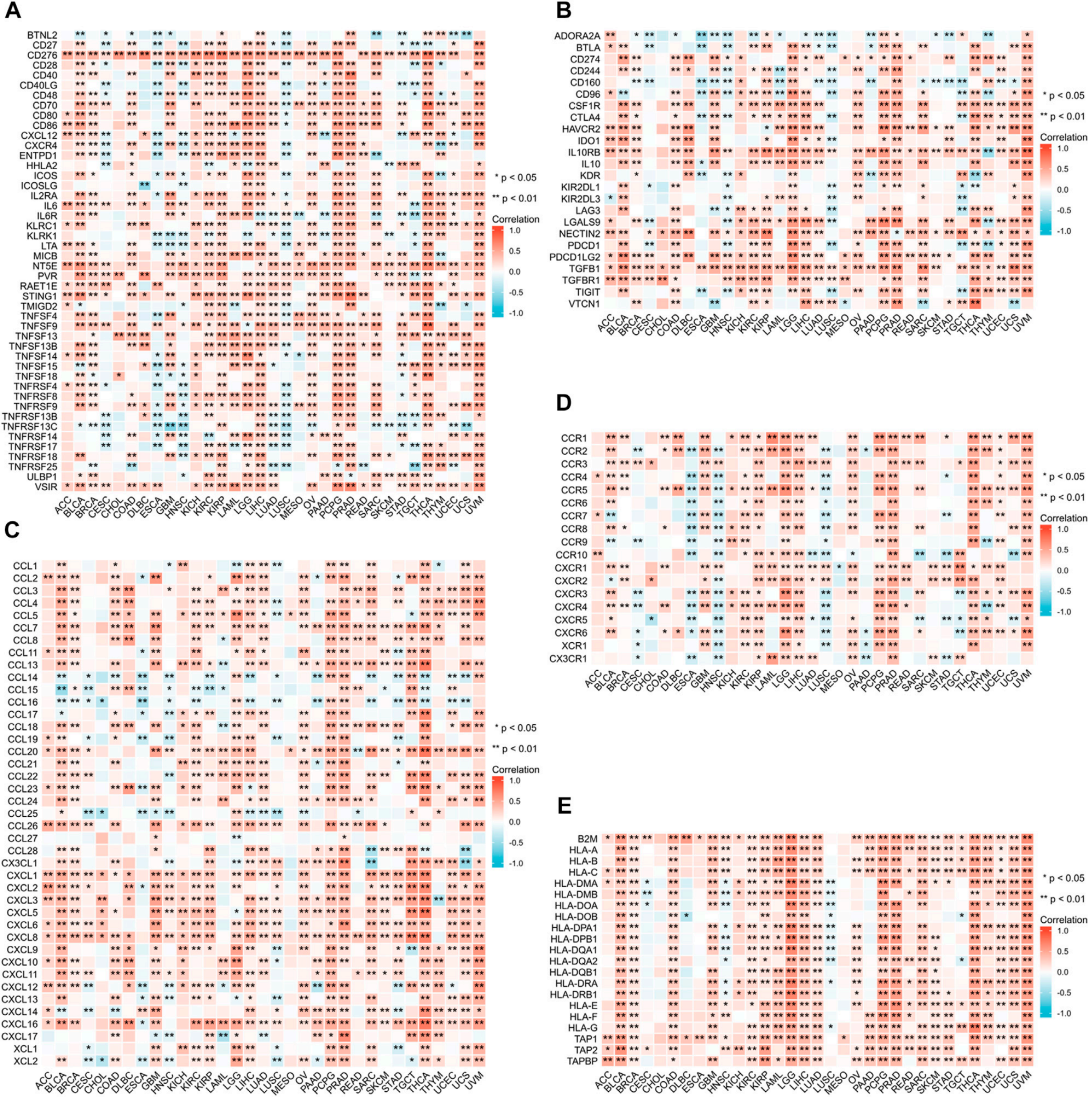
Relationship between ANXA2 expression and immunomodulators. **(A)** Immune-activating genes, **(B)** Immune-suppressing genes, **(C)** Chemokine ligands, **(D)** Chemokine receptors, **(E)** MHC genes. *p < .05 and **p < .01.

We conducted a gene co-expression analysis in 33 types of cancer exploring the correlation between ANXA2 expression and immunomodulators such as immune-activating genes, immune-suppressing genes, chemokine ligands, chemokine receptors and MHC genes (Figures 7A–E). ANXA2 was variably associated with immunomodulators studied in all the tumors (*p* < .05) and positively associated with most immunomodulators in 30 types of cancer (except CESC, HNSC and LUSC).

### Relationship between ANXA2 gene expression and DNA methylation and genetic alteration

**FIGURE 8:**
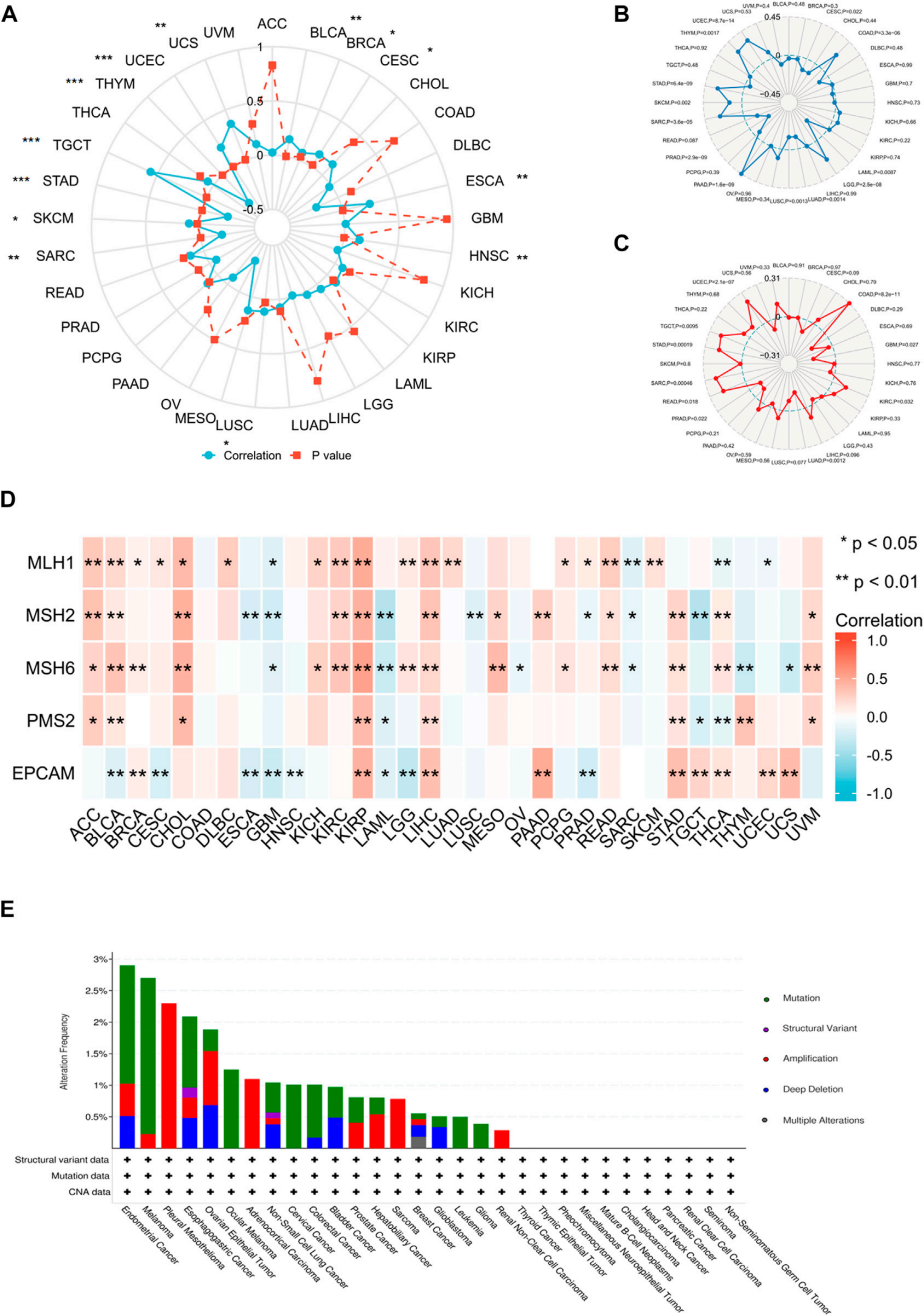
Relationship between ANXA2 gene expression and DNA methylation and genetic alteration in pan-cancer. **(A)** DNA methylation, **(B)** TMB, **(C)** MSI, **(D)** MMR, **(E)** Genetic variations. *p < .05, **p < .01 and ***p < .001.

We investigated the correlation between the ANXA2 expression and DNA methylation in pan-cancer (Table 1). The expression of ANXA2 positively correlated with DNA methylation in BLCA (r = .162, *p* = .001), BRCA (r = .075, *p* = .035), CESC (r = .135, *p* = .019), ESCA (r = .257, *p* = .001), HNSC (r = .146, *p* = .001), LUSC (r = .114, *p* = .029), SKCM (r = .102, *p* = .027), TGCT (r = .567, *p* < .001), UCEC (r = .210, *p* < .001) and UCS (r = .363, *p* = .006) and negatively correlated with DNA methylation in sarcoma (SARC) (r = −.198, *p* = .001), STAD (r = −.244, *p* < .001) and THYM (r = −.355, *p* < .001) (Figure 8A).

**TABLE 1:** Relationship between ANXA2 expression and DNA methylation in pan-cancer.

Type of cancer	Correlation	*p*-value
ACC	.025	.829
BLCA	.162	.001
BRCA	.075	.035
CESC	.135	.019
CHOL	.138	.420
COAD	−.022	.710
DLBC	−.221	.130
ESCA	.257	.001
GBM	.010	.943
HNSC	.146	.001
KICH	−.030	.811
KIRC	.080	.157
KIRP	.108	.076
LAML	.056	.554
LGG	.033	.461
LIHC	−.013	.809
LUAD	.075	.111
LUSC	.114	.029
MESO	.130	.231
OV	−.321	.498
PAAD	−.079	.298
PCPG	.123	.102
PRAD	−.070	.119
READ	.131	.199
SARC	−.198	.001
SKCM	.102	.027
STAD	−.244	<.001
TGCT	.567	<.001
THCA	.064	.150
THYM	−.355	<.001
UCEC	.210	<.001
UCS	.363	.006
UVM	.116	.306

The abbreviations of cancer types in TCGA: ACC, adrenocortical carcinoma; BLCA, bladder urothelial carcinoma; BRCA, breast invasive carcinoma; CESC, cervical squamous cell carcinoma and endocervical adenocarcinoma; CHOL, cholangiocarcinoma; COAD, colon adenocarcinoma; DLBC, lymphoid neoplasm diffuse large B-cell lymphoma; ESCA, esophageal carcinoma; GBM, glioblastoma multiforme; HNSC, head and neck squamous cell carcinoma; KICH, kidney chromophobe; KIRC, kidney renal clear cell carcinoma; KIRP, kidney renal papillary cell carcinoma; LAML, acute myeloid leukemia; LGG, brain lower grade glioma; LIHC, liver hepatocellular carcinoma; LUAD, lung adenocarcinoma; LUSC, lung squamous cell carcinoma; MESO, mesothelioma; OV, ovarian serous cystadenocarcinoma; PAAD, pancreatic adenocarcinoma; PCPG, pheochromocytoma and paraganglioma; PRAD, prostate adenocarcinoma; READ, rectum adenocarcinoma; SARC, sarcoma; SKCM, skin cutaneous melanoma; STAD, stomach adenocarcinoma; TGCT, testicular germ cell tumors; THCA, thyroid carcinoma; THYM, thymoma; UCEC, uterine corpus endometrial carcinoma; UCS, uterine carcinosarcoma; UVM, uveal melanoma.

We further investigated the correlation between ANXA2 expression and TMB, MSI and MMR in 33 types of cancer. The ANXA2 expression positively correlated with TMB in eight types of cancer (all r > 0): COAD (*p* < .001), LGG (*p* < .001), PAAD (*p* < .001), SARC (*p* < .001), SKCM (*p* = .002), STAD (*p* < .001), THYM (*p* = .0017) and UCEC (*p* < .001). The ANXA2 expression negatively correlated with TMB in five types of cancer (all r < 0): CESC (*p* = .022), LAML (*p* = .0087), LUAD (*p* = .0014), LUSC (*p* = .0013), and PRAD (*p* < .001) (Figure 8B). The expression of ANXA2 positively correlated with MSI in seven types of cancer (all r > 0): COAD (*p* < .001), KIRC (*p* = .032), READ (*p* = .018), SARC (*p* < .001), STAD (*p* < .001), TGCT (*p* = .0095) and UCEC (*p* < .001). The ANXA2 expression negatively correlated with MSI in three types of cancer (all r < 0): GBM (*p* = .027), LUAD (*p* = .0012) and PRAD (*p* = .022) (Figure 8C). The ANXA2 expression was variably associated with five MMR genes (MLH1, MSH2, MSH6, PMS2 and EPCAM) in 32 types of cancer and was closely correlated with MMR in BLCA, KIRP and LIHC (Figure 8D). ANXA2 showed the highest frequency of variation in UCEC (2.9%). Gene mutations were the most common type of genetic variations. The highest mutation frequency of ANXA2 was reported in SKCM (2.48%) (Figure 8E).

### GSEA

**FIGURE 9:**
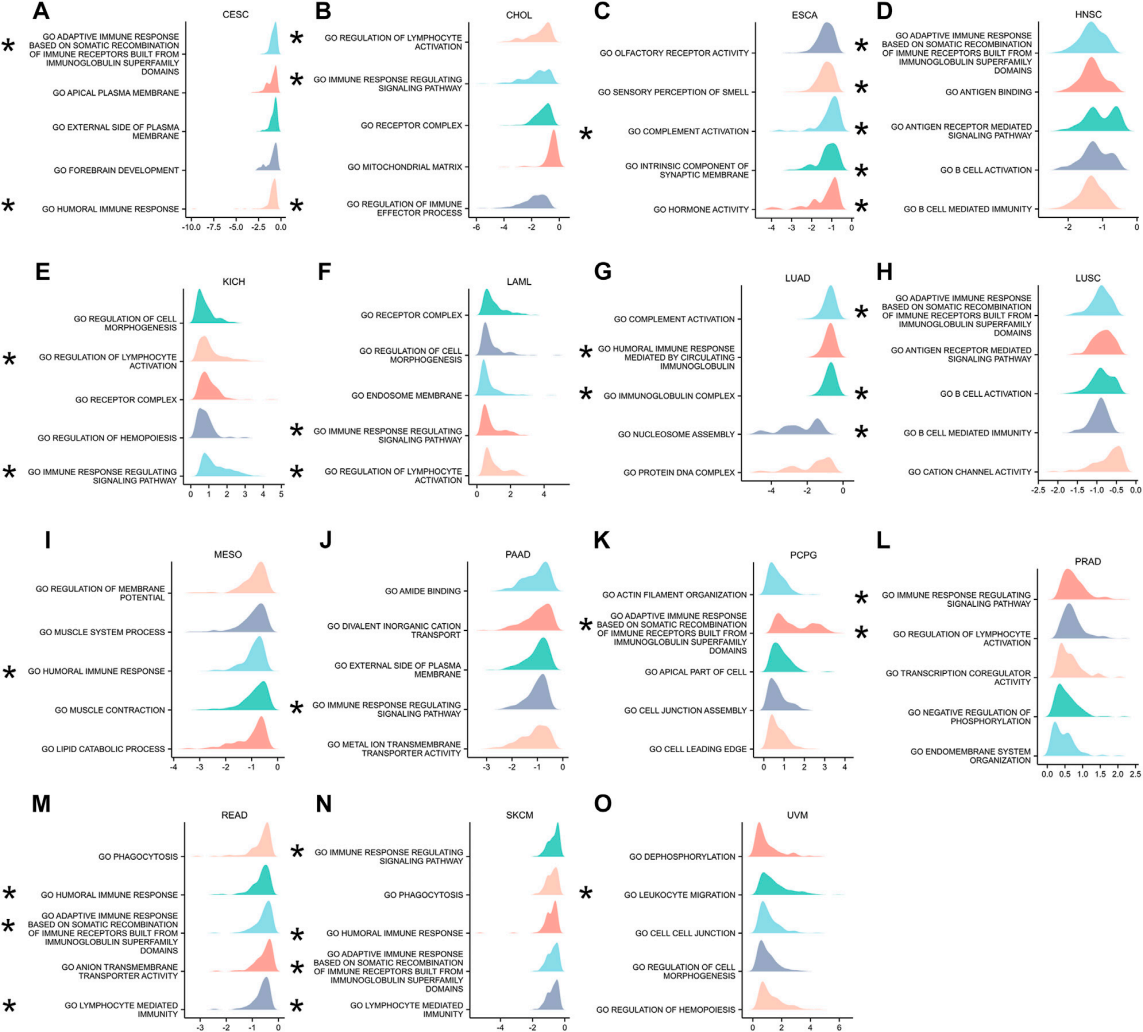
GSEA containing immune-related pathways. **(A–O)** The top five GSEA terms identified in specific tumor types.

The GSEA was conducted to further explore the ANXA2-related pathways in tumorigenesis and tumor development. ANXA2 was associated with immune-related pathways in CESC, CHOL, ESCA, HNSC, KICH, LAML, LUAD, LUSC, MESO, PAAD, PCPG, PRAD, READ, SKCM and UVM. In CESC, ANXA2-related immune pathway were adaptive immune response based on somatic recombination of immune receptors bulit from immunoglobulin superfamily domains and humoral immune response (Figure 9A). In CHOL, ANXA2-related immune pathways were regulation of lymphocyte activation, immune response regulating signaling pathway and regulation of immune effector process (Figure 9B). In ESCA, ANXA2-related immune pathway was complement activation (Figure 9C). In HNSC, ANXA2-related immune pathway were adaptive immune response based on somatic recombination of immune receptors bulit from immunoglobulin superfamily domains, antigen binding, antigen receptor mediated signaling pathway, B cell activation and B cell mediated immunity (Figure 9D). In KICH, LAML and PRAD, ANXA2-related immune pathway were regulation of lymphocyte activation and immune response regulating signaling pathway (Figures 9E,F,L). In LUAD, the ANXA2-related immune pathway were humoral immune response mediated by circulating immunoglobulin and immunoglobulin complex (Figure 9G). In LUSC, ANXA2-related immune pathway were adaptive immune response based on somatic recombination of immune receptors bulit from immunoglobulin superfamily domains, B cell activation and B cell mediated immunity (Figure 9H). In MESO, the ANXA2-related immune pathway was the humoral immune response (Figure 9I). In PAAD, ANXA2-related immune pathway was immune response regulating signaling pathway (Figure 9J). In PCPG, ANXA2-related immune pathway was adaptive immune response based on somatic recombination of immune receptors bulit from immunoglobulin superfamily domains (Figure 9K). In READ, ANXA2 related immune pathway were humoral immune response, adaptive immune response based on somatic recombination of immune receptors bulit from immunoglobulin superfamily domains and lymphocyte mediated immunity (Figure 9M). In SKCM, ANXA2-related immune pathways were immune response regulating signaling pathway, humoral immune response, adaptive immune response based on somatic recombination of immune receptors bulit from immunoglobulin superfamily domains, humoral immune response and lymphocyte mediated immunity (Figure 9N). In UVM, the ANXA2-related immune pathway was leukocyte migration (Figure 9O). The above-mentioned immune-related pathways were not observed in other tumors (Supplementary Figure S5).

## Discussion

Annexins are a large family of calcium-dependent membrane-binding proteins that associate with membrane lipids and cytoskeletal components (Dallacasagrande and Hajjar, 2020). The annexin superfamily consists of five subfamilies (A, B, C, D and E), and twelve annexins in vertebrate cells constitute the annexin A family (A1-A11 and A13) (Zhao et al., 2022). ANXA2 is a widely studied member of the annexin superfamily and is expressed in various cell types, including dendritic cells, monocytes, macrophages, bone marrow cells, epithelial cells, endothelial cells, neurons and tumor cells (Wang and Lin, 2014; Huang et al., 2022). ANXA2 normally exists as a monomer or heterotetramer. The ANXA2 monomer is located in the cytoplasm while the ANXA2 heterotetramer consists of two ANXA2 monomers and two S100A10 protein monomers located in the membrane (Zhang et al., 2012a). ANXA2 is abnormally expressed in a variety of tumors, such as glioma (Ma et al., 2021b), cervical cancer (Wang et al., 2021), LIHC (Yu et al., 2007), triple-negative breast cancer (Gibbs et al., 2020) and nasopharyngeal carcinoma (Chen et al., 2018), and can serve as a prognostic marker in these tumors. To the best of our knowledge, this is the first reasearch performing a detailed bioinformatics analysis of ANXA2 in pan-cancer, revealing its prognostic value.

In this study, we evaluated the ANXA2 expression in 33 types of cancer and corresponding normal tissues from TCGA. The ANXA2 expression was high in 12 types of cancer and low in four types of cancer. Combining together the TCGA and GTEx databases, 23 types of cancer showed high ANXA2 expression, while five showed low expression. ANXA2 was upregulated in most types of cancer, which allowed it to function more fully as an potential oncogene. It showed that ANXA2 played a general role in most types of cancer, demonstrating its importance in tumor development promotion and led to poor prognosis through epithelial-mesenchymal transition, cancer drug resistance and other pathways (Wang et al., 2019; Huang et al., 2022). We demonstrated a differential protein expression of ANXA2 in five types of cancer, including COAD, GBM, LIHC, PRAD and STAD. It is worth noting that the ANXA2 expression in BRCA was lower than the corresponding normal tissues by using the TCGA database. When the TCGA and GTEx databases were combined together, the findings were opposite. Previous studies suggested that the ANXA2 expression was higher in breast cancer than corresponding normal tissues (Zhao et al., 2020; Abdelraouf et al., 2022). These studies were all concluded through experimental validation and did not involve bioinformatics analysis. The contradiction between the experimental results and the bioinformatics analysis results needs to be verified by deeper experiments or more datasets, which will enable us to better understand the role of ANXA2 in breast cancer. In both analysis methods, the expression of ANXA2 in KICH and PRAD was lower than that in corresponding normal tissues. The low expression of ANXA2 in PRAD has been widely proved (Anselmino et al., 2020). In addition, we found that ANXA2 was mainly located in the cell membrane and cytoplasm, with a small amount in the nucleus, which was consistent with a previous studies (Xu et al., 2015). Differential expression of ANXA2 might contribute to the occurrence and development of various cancers (Yang et al., 2022).

Several genetic biomarkers have been used to predict tumor prognoses (Karamichalis et al., 2016), and in a sense, they are even more reliable than histopathological diagnoses (Hu et al., 2021). This study examined the diagnostic and prognostic efficacy of ANXA2 in 33 types of cancer. ANXA2 showed a high diagnostic efficacy in 12 types of cancer, including CHOL, GBM, KICH, KIRP, LIHC, OV, PAAD, PRAD, READ, STAD, TGCT and THYM. Consistently with our findings, previous studies demonstrated a diagnostic value of ANXA2 in LIHC (Zhang et al., 2012b), PAAD (Zhang et al., 2022) and PRAD (Li et al., 2021). In the survival analysis, the expression of ANXA2 correlated with OS, PFI and DSS in several types of tumors. The expression of ANXA2 positively correlated with PFI in USEC and negatively correlated with OS, PFI and DSS in several other tumors, demonstating that high ANXA2 expression was associated with poor prognosis in many tumors. Similarly, the expression of ANXA2 negatively correlated with OS, PFI and DSS in seven types of cancer, including BLCA, HNSC, LGG, LUAD, MESO, PAAD and UVM. The ANXA2 expression was associated with the clinical T stage of five types of cancer, the clinical N stage of five types of cancer and the clinical M stage of four types of cancer, supporting a potential prognostic value in these tumors. The diagnostic and prognostic abilities of ANXA2 in various cancers suggest its potential as a marker for the early diagnosis.

The tumor microenvironment (TME) is the ecosystem that surrounds a tumor inside the body and includes resident stromal cells, immune cells, the extracellular matrix and blood vessels. The TME is involved in tumor development and significantly influences therapeutic response and clinical outcome (Wu and Dai, 2017). Understanding the tumor immune microenvironment is relevant for identifying immunomodulators involved in cancer progression and developing targeted immunotherapies (Ren et al., 2021). We explored the correlation between ANXA2 expression and immune infiltration in 33 types of cancer. The ANXA2 expression was significantly associated with the infiltration of six immune cells (B cells, dendritic cells, macrophages, neutrophils, NK cells and T cells) in BLCA, PCPG, PRAD, TGCT and THCA, indicating that ANXA2 might influence the immune responses in these six types of cancer. When 24 types of immune cells were investigated, ANXA2 expression variably correlated with them in 32 types of cancer (except CHOL). In addition, ANXA2 expression was associated with StromalScore, ImmuneScore and ESTIMATEScore in different tumors, suggesting that ANXA2 was strongly related to the immune microenvironment in multiple cancers. In cancer, ANXA2 usually plays a pro-inflammatory role, and the excessive angiogenesis maintained by ANXA2 under inflammatory conditions may induce tissue damage and lead to poor prognosis (Dallacasagrande and Hajjar, 2020). In the era of stratified medicine, it is increasingly vital to identify new immune-related biomarkers (Chen et al., 2019). Our study provides a theoretical support for the application of ANXA2 in cancer immunotherapy.

Immune checkpoints are regulators of the immune system that are crucial to maintaining self-tolerance and preventing autoimmunity (Pardoll, 2012). At the same time, immune checkpoints are one of the ways for cancer cells to camouflage (Li et al., 2019). In the field of cancer treatment, immunocheckpoint blocking has become one of the most important immunotherapeutic methods, which can trigger tumor cell killing mechanisms by activating tumor antigen specific T cell reaction (Li et al., 2019; Ren et al., 2021). Inhibition of the programmed cell death protein 1 (PD-1) and cytotoxic T-lymphocyte-associated protein 4 (CTLA-4) immune checkpoints has led to new immunotherapies against cancer (Rotte, 2019). In this study, we found that ANXA2 variably correlated with 47 immune checkpoints in 33 types of cancer. The most common correlation was observed with CD276, a costimulatory/coinhibitory immunoregulatory protein that can inhibit the proliferation of T cells (Liu et al., 2021). Upregulation of CD276 promoted the immune escape of tumor cells and reduced secretion of interleukin-2 (IL-2), tumor necrosis factor-alpha (TNF-α) and other cytokines (Ma et al., 2016). CD276 is a potential target for cancer immunotherapy (Picarda et al., 2016). However, the relationship between CD276 and ANXA2 is unknown. CD276 promoted tumor metastasis by participating in epithelial mesenchymal transformation (EMT) (Kang et al., 2015; Liu et al., 2021). We speculate that he EMT process may be the link between ANXA2 and CD276, and CD276 may be the key factor for breakthrough in cancer immunotherapy targeting ANXA2, which needs more research to prove. Of interest, ANXA2 expression in CHOL was associated with only two immune checkpoints, CD276 and TNFSF18. The results of immune infiltration analysis showed although that ANXA2 expression did not correlate with infiltration of 24 types of immune cells in CHOL, its expression was highly related to expression of immunomodulators such as immune activating genes, immune suppressing genes, chemokine ligands, chemokine receptors and MHC genes in 33 types of cancer. Therefore, ANXA2 is a potential immune-related marker and a promising immunotherapy target in pan-cancer.

Methylation is a form of epigenetic modification that plays a significant role in gene regulation (Fang et al., 2016). An abnormal DNA methylation usually occurs in the promoter region of several transcription factors involved in cancer (Klutstein et al., 2016). In this study, the expression of ANXA2 was associated with DNA methylation in many tumors. Specifically, ANXA2 expression was positively correlated with DNA methylation in BLCA, BRCA, CESC, ESCA, HNSC, LUSC, SKCM, TGCT, UCEC and UCS and negatively correlated with DNA methylation in SARC, STAD and THYM. Previous studies identified ANXA2 as a unique methylation-dependent positive regulator of the GBM mesenchymal subtype, suggesting its potential diagnostic ability (Kling et al., 2016). Exploring the relationship between ANXA2 and DNA methylation will contribute to elucidating the role of ANXA2 in cancer development.

The TMB represents the number of non-inherited mutations per million bases of investigated genomic sequence (Chan et al., 2019). The MSI represents a predisposition to mutation resulting from an impaired DNA mismatch repair (Baretti and Le, 2018). Both TMB and MSI are predictive biomarkers for cancer patients receiving immunotherapy. Patients with high TMB or MSI are more likely to benefit from immunotherapy in the long term (Chan et al., 2019; Yamamoto et al., 2020). In this study, ANXA2 significantly correlated with TMB and MSI in several tumors (such as COAD, STAD and UCEC), and most of them were positively correlated. In most tumors, ANXA2 was positively associated with five MMR genes (MLH1, MSH2, MSH6, PMS2 and EPCAM), suggesting the importance of ANXA2 in DNA repair. These findings contribute to revealing the role of ANX2 in cancer-related genetic changes, providing guidance for a future application in immunotherapy.

The GSEA was conducted to further understand the role of ANXA2 in tumors. ANXA2 was significantly related to immune pathways in CESC, CHOL, ESCA, GBM, HNSC, KICH, LAML, LUAD, LUSC, MESO, PAAD, PCPG, READ, SKCM and UVM. The immune pathways included humoral immune responses, complement activation and B cell-mediated immunity. Previous studies found that ANXA2 played an anti-inflammatory role in response to an injury or an infection. The anti-inflammatory effect maintained the integrity of blood vessels and prevented the activation of inflammatory factors (Dallacasagrande and Hajjar, 2020). The ANXA2 monomer on the cell membrane might be a new ligand for the complement cascade (Martin et al., 2012). These results revealed the main roles of ANXA2 in different tumors, emphasizing the importance of ANXA2 in immune regulation.

However, it should be pointed out that our work is a retrospective study using multiple public databases. The application of bioinformatics technology can provide us with the valuable theoretical basis of the pathogenic role of ANXA2 in pan-cancer. In addition, further clarification of the relationship between ANXA2 and patient prognosis requires rigorous experimental validation and more multicenter prospective studies, guiding ANXA2 application in the treatment of cancer patients.

## Conclusion

In conclusion, we demonstrated the differential expression of ANXA2 between tumor and normal tissues, and ANXA2 could be used as a prognostic biomarker for various tumors. The ANXA2 expression was variably related to infiltration of different immune cells, immune checkpoints, DNA methylation, TMB and MSI, revealing the important role of ANXA2 in immune regulation of various tumors and its potential application as an immunotherapeutic target. Further research on ANXA2 may lead to new breakthroughs for cancer diagnosis and immunotherapy, and further improve the prognosis of cancer patients.

## Data Availability

The original contributions presented in the study are included in the article/Supplementary Material, further inquiries can be directed to the corresponding author.
