# Supplementary material for: ANXA2 is a potential biomarker for cancer prognosis and immune infiltration: A systematic pan-cancer analysis
Source: Front Genet. 2023 Jan 12;14:1108167. doi: 10.3389/fgene.2023.1108167 (PMC9877333; doi:10.3389/fgene.2023.1108167)
Supplement: Supplementary file 1 [file Table1.DOCX]

**Supplementary Table 1.** The sample sizes for each comparison in differential expression analysis.

| **Type of cancer** | **TCGA** | | **TCGA+GTEx** | | **Paired samples** | |
| --- | --- | --- | --- | --- | --- | --- |
|  | **Normal** | **Tumor** | **Normal** | **Tumor** | **Normal** | **Tumor** |
| ACC | 0 | 77 | 128 | 77 | 0 | 0 |
| BLCA | 19 | 407 | 28 | 407 | 19 | 19 |
| BRCA | 113 | 1099 | 292 | 1099 | 112 | 112 |
| CESC | 3 | 306 | 13 | 306 | 0 | 0 |
| CHOL | 9 | 36 | 9 | 36 | 9 | 9 |
| COAD | 41 | 290 | 349 | 290 | 26 | 26 |
| DLBC | 0 | 47 | 444 | 47 | 0 | 0 |
| ESCA | 13 | 182 | 666 | 182 | 13 | 13 |
| GBM | 5 | 166 | 1157 | 166 | 0 | 0 |
| HNSC | 44 | 520 | 44 | 520 | 43 | 43 |
| KICH | 25 | 66 | 53 | 66 | 25 | 25 |
| KIRC | 72 | 531 | 100 | 531 | 72 | 72 |
| KIRP | 32 | 289 | 60 | 289 | 32 | 32 |
| LAML | 0 | 173 | 70 | 173 | 0 | 0 |
| LGG | 0 | 523 | 1152 | 523 | 0 | 0 |
| LIHC | 50 | 371 | 160 | 371 | 50 | 50 |
| LUAD | 59 | 515 | 347 | 515 | 58 | 58 |
| LUSC | 50 | 498 | 338 | 498 | 50 | 50 |
| MESO | 0 | 87 | 0 | 87 | 0 | 0 |
| OV | 0 | 427 | 88 | 427 | 0 | 0 |
| PAAD | 4 | 179 | 171 | 179 | 4 | 4 |
| PCPG | 3 | 182 | 3 | 182 | 0 | 0 |
| PRAD | 52 | 496 | 152 | 496 | 52 | 52 |
| READ | 10 | 93 | 318 | 93 | 6 | 6 |
| SARC | 2 | 262 | 2 | 262 | 0 | 0 |
| SKCM | 1 | 439 | 813 | 439 | 0 | 0 |
| STAD | 36 | 414 | 210 | 414 | 33 | 33 |
| TGCT | 0 | 154 | 165 | 154 | 0 | 0 |
| THCA | 59 | 512 | 338 | 512 | 59 | 59 |
| THYM | 2 | 119 | 446 | 119 | 0 | 0 |
| UCEC | 23 | 181 | 101 | 181 | 7 | 7 |
| UCS | 0 | 57 | 78 | 57 | 0 | 0 |
| UVM | 0 | 79 | 0 | 79 | 0 | 0 |
