# Supplementary material for: ANXA2 is a potential biomarker for cancer prognosis and immune infiltration: A systematic pan-cancer analysis
Source: Front Genet. 2023 Jan 12;14:1108167. doi: 10.3389/fgene.2023.1108167 (PMC9877333; doi:10.3389/fgene.2023.1108167)
Supplement: Supplementary file 2 [file Image1.pdf]

Supplementary Figures

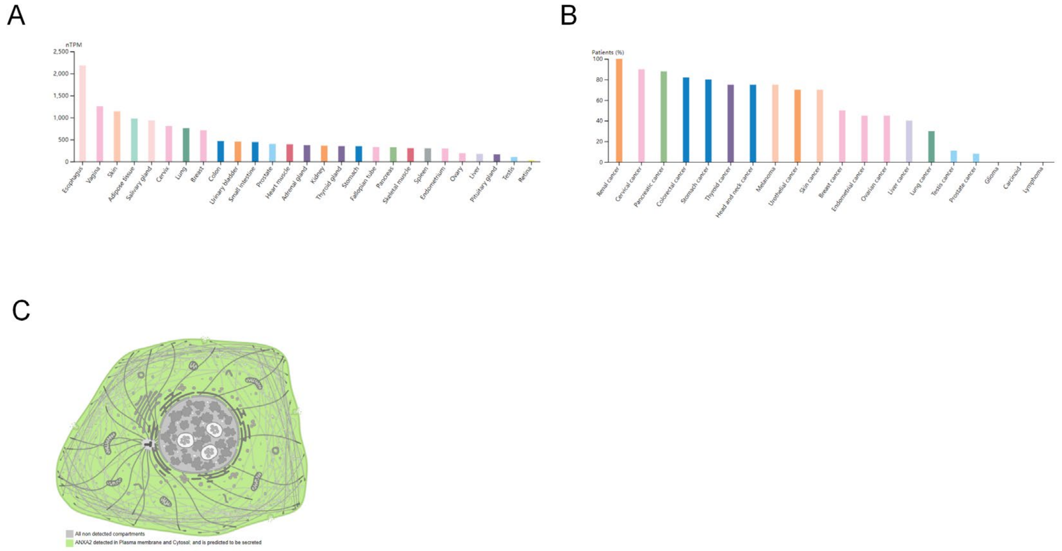

Supplementary Figure 1. The protein expression and intracellular localization of ANXA2. (A) The protein expression of ANXA2 in 27 types of normal tissues. (B) The protein expression of ANXA2 in 20 cancers. (C) Intracellular localization of ANXA2 protein.

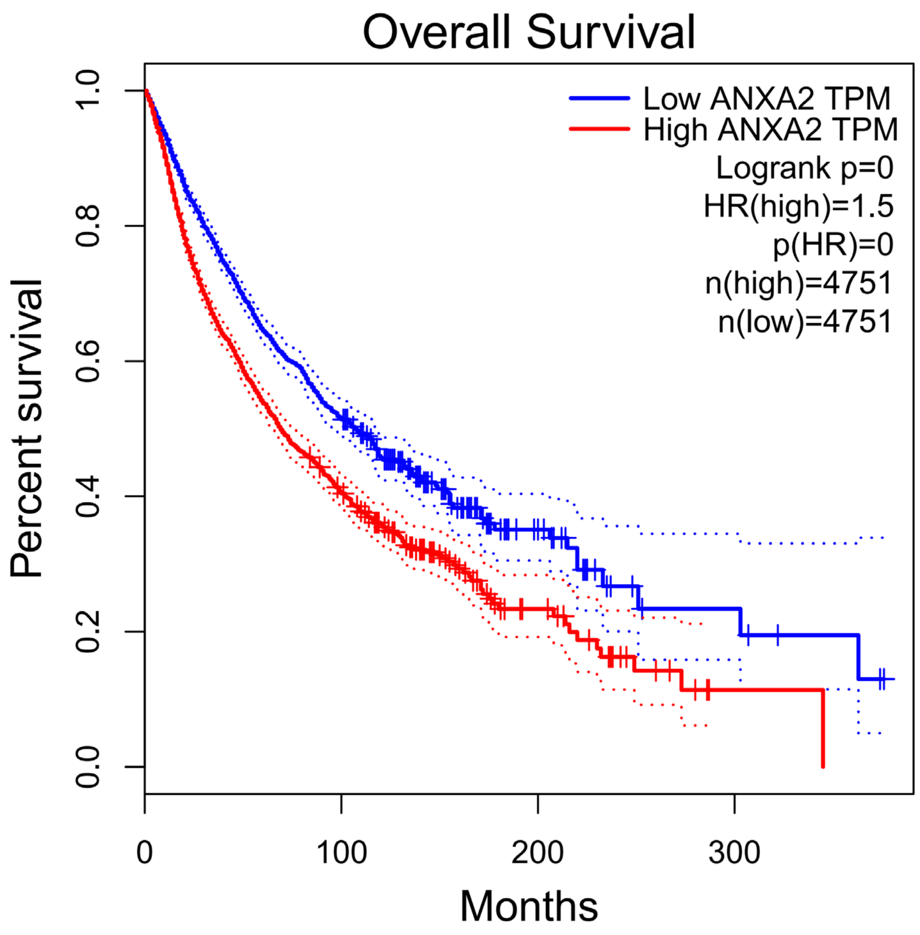

Supplementary Figure 2. The pan-cancer survival analysis of ANXA2 gene based on the GEPIA database.

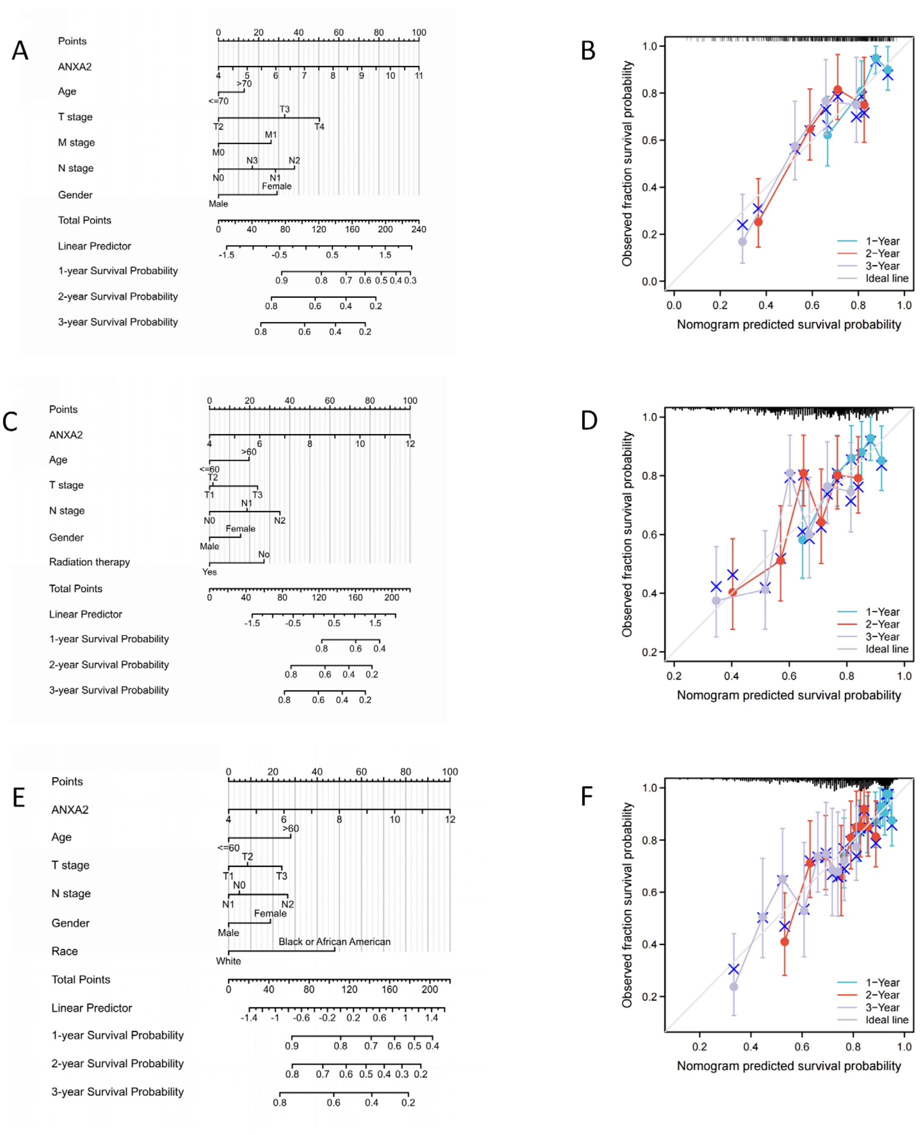

Supplementary Figure 3. Nomograms and corresponding calibration curves. (A-B) Nomograms for BLCA and corresponding calibration curves. (C-D) Nomograms for HNSC and corresponding calibration curves. (E-F) Nomograms for LUAD and corresponding calibration curves.

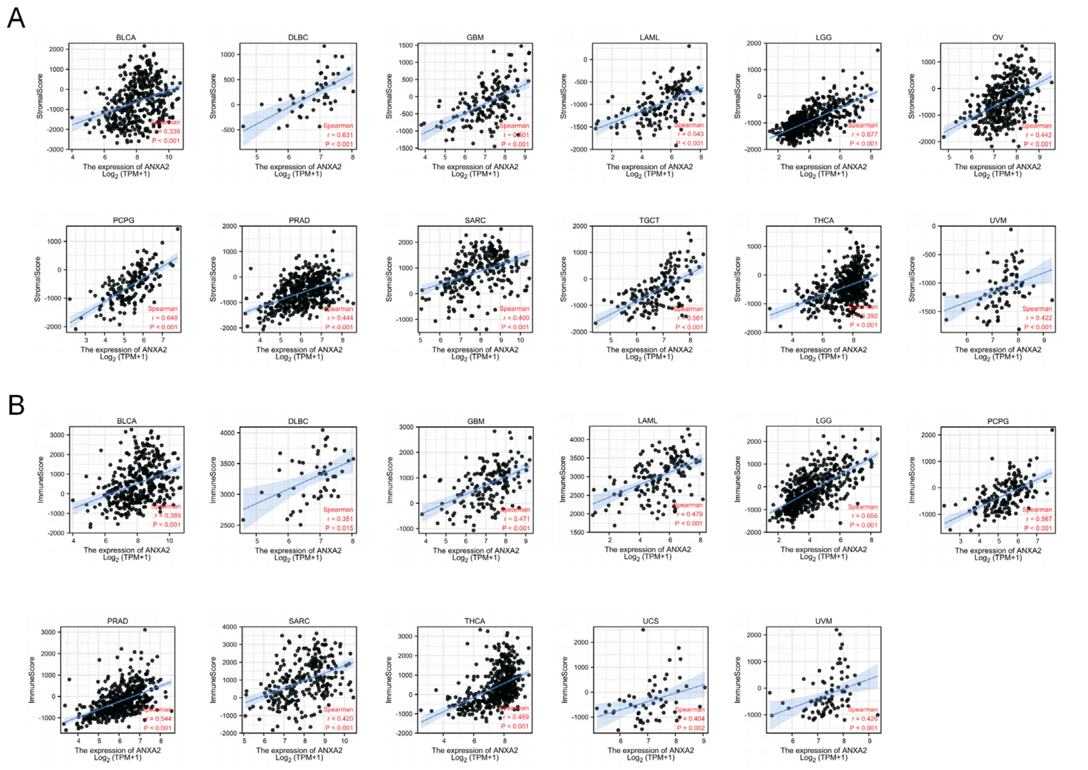

Supplementary Figure 4. Relationship between ANXA2 gene expression and StromalScore and ImmuneScore in pan-cancer. (A) ANXA2 expression significantly correlated with the StromalScore in 12 types of cancer. (B) ANXA2 expression significantly correlated with the ImmuneScore in 11 types of cancer.

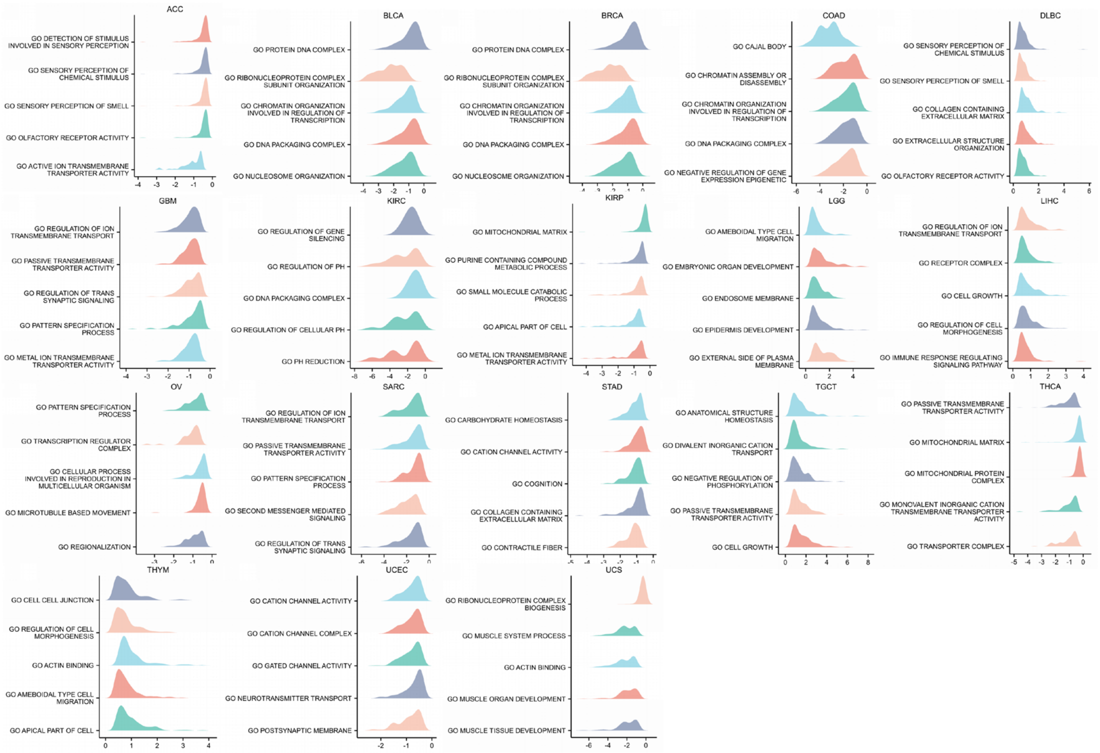

Supplementary Figure 5. GSEA of ANXA2.
